# Integrated role of human thymic stromal cells in hematopoietic stem cell extravasation

**DOI:** 10.1002/btm2.10454

**Published:** 2022-11-17

**Authors:** Sara A. Watson, Yousef Javanmardi, Luca Zanieri, Somayeh Shahreza, Roberta Ragazzini, Paola Bonfanti, Emad Moeendarbary

**Affiliations:** ^1^ Department of Mechanical Engineering UCL London UK; ^2^ Epithelial Stem Cell Biology and Regenerative Medicine Lab The Francis Crick Institute London UK; ^3^ Institute of Immunity and Transplantation Division of Infection & Immunity, UCL London UK

**Keywords:** drug discovery and development, lymphoid progenitors, microfluidic devices, organ‐mimetic systems, regenerative medicine, thymus on a chip, tissue engineering

## Abstract

The human thymus is the site of T‐cell maturation and induction of central tolerance. Hematopoietic stem cell (HSC)‐derived progenitors are recruited to the thymus from the fetal liver during early prenatal development and from bone marrow at later stages and postnatal life. The mechanism by which HSCs are recruited to the thymus is poorly understood in humans, though mouse models have indicated the critical role of thymic stromal cells (TSC). Here, we developed a 3D microfluidic assay based on human cells to model HSC extravasation across the endothelium into the extracellular matrix. We found that the presence of human TSC consisting of cultured thymic epithelial cells (TEC) and interstitial cells (TIC) increases the HSC extravasation rates by 3‐fold. Strikingly, incorporating TEC or TIC alone is insufficient to perturb HSC extravasation rates. Furthermore, we identified complex gene expressions from interactions between endothelial cells, TEC and TIC modulates the HSCs extravasation. Our results suggest that comprehensive signaling from the complex thymic microenvironment is crucial for thymus seeding and that our system will allow manipulation of these signals with the potential to increase thymocyte migration in a therapeutic setting.

## 1 INTRODUCTION

The thymus is the primary lymphoid organ where hematopoietic progenitors are instructed to develop into T cells during thymopoiesis. Thymus seeding progenitors (TSP) are recruited from the pool of hematopoietic stem cells (HSC) in the fetal liver^1^ and bone marrow^2^ during early development and postnatal life, respectively. While the developing thymocytes represent the majority of the thymus cellularity^3^, ^4^ thymus stromal cells (TSC) including thymic epithelial cells (TEC), thymic interstitial cells (TIC), and endothelial cells (EC) comprise the 3D framework in which thymocytes mature.

The overall process of TSP entrance into the thymus has been poorly described in humans due to a lack of appropriate models, though extrapolation from mouse data suggests that chemokines secreted by TEC, such as CCL25^5^, ^6^ and CXCL12,^7^, ^8^ identified as ligands to CCR9 and CXCR4 receptors, respectively, as well as the expression of P‐selectin will play a critical role in TSP migration and extravasation.^9^ Transendothelial migration occurs most frequently within postcapillary venules^10^ and may transpire in one of two ways.^11^ In transcellular extravasation, a leukocyte passes through an endothelial cell, while the biomechanically favorable paracellular extravasation occurs between endothelial cell junctions.^12^, ^13^ The first early T‐cell progenitors (ETP) are detected in the human fetal thymus as early as Week 5^14^ though significant seeding is not seen until Week 8.^15^ By Week 12 of embryonic development, mature T cells have begun to leave the thymus, continuing through development and postnatal life.^4^ Crucial results from HSC transplantation^16^, ^17^ and gene therapies^18^ have shown that transplanted progenitors are long lived and can sustain immune cell production postintervention, suggesting that TSP continue to migrate to the postnatal thymus. Recent transcriptomic analyses of thymocytes from postnatal human thymi have identified two distinct populations of TSP within the thymus characterized by their expression of markers such as CCR7, CCR9, and CD10 (TSP1) and ITGβ7 and CD7 expression (TSP2) with the latter representing a “primed” state.^19^ While ETP represent a very small fraction of the total hematopoietic cells within the thymus, they maintain a spectrum of lineage genes and can give rise to both myeloid and lymphoid cells including plasmacytoid dendritic cells, NK T cells, and innate lymphoid cells.^20^, ^21^ As the cross‐talk between the developing thymocytes and the TEC is crucial for both T cell and epithelial development^22^, ^23^, ^24^ and Notch signaling is critical for T lineage commitment,^25^, ^26^, ^27^, ^28^ the interactions between the TSP and other TSC, such as EC and TIC, may be similarly crucial for progenitor recruitment and commitment.

Significant efforts have been made to partly recapitulate the complexity of in vivo conditions using in vitro physiological systems. Recent advances in thymus reconstruction have allowed for long‐term expansion of progenitors and functional reconstitution of the thymus.^29^ However, the time scale and cellular number required make molecular insights difficult to interrogate in a systematic manner. Microfluidic cell culture devices facilitate 3D cell culture and allow tissue and organ‐level functions to be assessed and recently have been utilized particularly well to mimic vascular formation and function.^30^, ^31^, ^32^, ^33^ High‐throughput analysis with minimal cell number requirements, spatiotemporally high‐resolution live microscopy, and retrieval of biological material are key advantages of microfluid‐based 3D culture systems compared to bioreactors or organotypic culturing. Several in vitro models have been developed to investigate cellular extravasation in other contexts, that is, cancer^34^, ^35^, ^36^, ^37^, ^38^, ^39^, ^40^ and immune cells extravasation.^41^, ^42^ The methods have so far focused on the innate extravasation potential of the cells themselves with less attention on the influence of the microenvironment in which this extravasation occurs. We have therefore sought to develop an assay that mimics the vasculature while also incorporating the thymic stroma to better understand the influence of thymus microenvironment on progenitor seeding and T‐cell development.

## 2 RESULTS

### 2.1 A 3D microfluidic device recapitulates HSC extravasation

**FIGURE 1:**
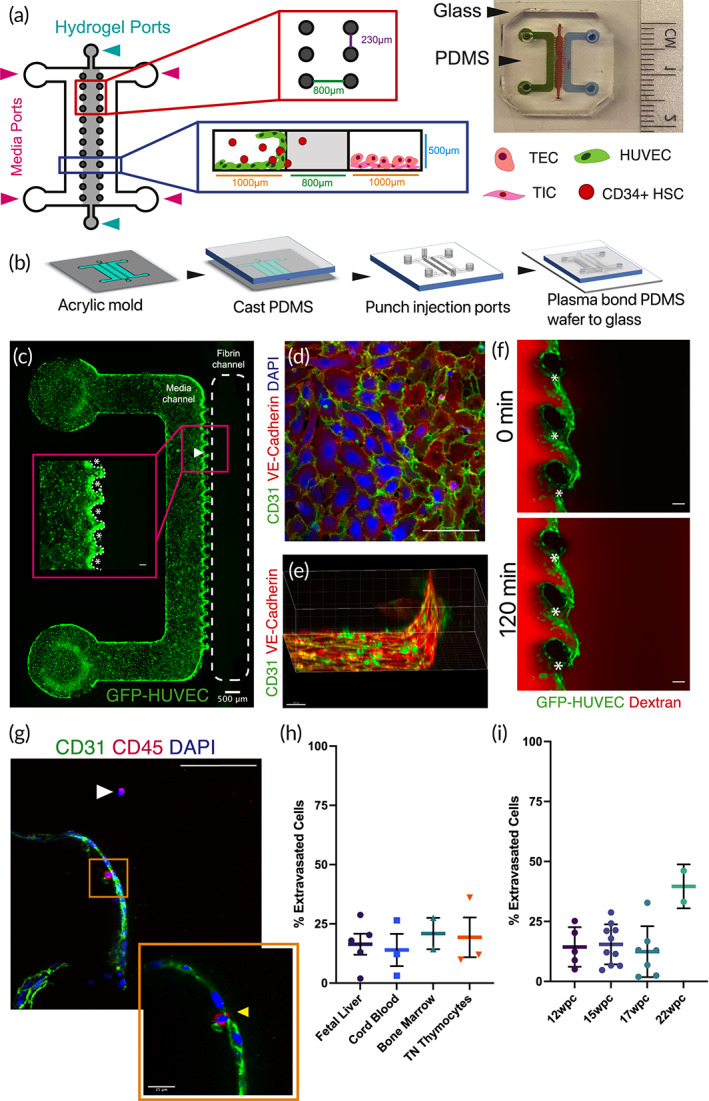
A microfluidic model of HSC extravasation. (a) Schematic of a polydimethylsiloxane (PDMS) microdevice consisting of three channels separated by PDMS posts distanced approximately 230 μm. The posts support the formation of the fibrin gel in the center channel. The channels are approximately 1 cm in length and 500 μm in height and the width of the central channel is 800 μm and the media channels 1 mm. The device allows formation of endothelial monolayer in between posts at the interface of left and hydrogel channels. Following the formation of monolayer CD34^+^ HSC can be seeded through left media channel. Other cell types, including TSC, can be seeded in the right channel. (b) Schematic of fabrication of PDMS chip using microfabricated mold. Following casting of the PDMS onto the mold, injection ports were created by punching holes into the PDMS with biopsy punches (2 mm for media ports and 1 mm for hydrogel channel). Subsequently, PDMS wafer was bonded to the plasma treated glass. (c) Image of the whole devices seeded with GFP‐HUVEC in the left channel. Scale bar 500 μm. Inset shows human umbilical vein endothelial cells (HUVEC) covering on the PDMS posts and the fibrin gel. (d) Staining of HUVEC with VE‐cadherin (red) and CD31 (green) shows tight endothelial junctions on the bottom of the endothelial channel. (e) A 3D reconstruction of the HUVEC monolayer formed between two posts. Scale bar 30 μm. (f) Fluorescent dextran (red) perfused through left media channel and slowly diffused across the monolayer into fibrin gel indicating the tight barrier function of the monolayer. The PDMS posts are indicated with asterisks (*). (g) CD45^+^ HSC (red) extravasated (white arrow) or in the process of extravasating through the endothelial monolayer (yellow arrow, inset). Inset scale bar 25 μm. (h) Extravasation rates of CD34+ HSC from fetal liver, cord blood, and bone marrow, as well as CD3^−^/CD4^−^/CD8^−^ triple negative thymocytes from thymus. (i) Extravasation rates of CD34+ HSC from fetal liver from 12, 15, 17, 22 wpc. Scale bars 100 μm unless otherwise indicated and nuclei stained with DAPI (blue)

To model HSC extravasation in the thymic environment, we employed three channel polydimethylsiloxane (PDMS) microdevices and studied HSC transendothelial migration (Figure 1a). The central channel of the device, which has a volume of 0.4 mm^3^, was filled with a fibrin hydrogel that is biochemically inert, has sufficient strength to support human umbilical vein endothelial cells (HUVEC) monolayer formation, and is sufficiently porous for HSC (pores > > 1 μm) 3D migration.^43^ To recapitulate the lumen of a vessel, HUVEC were seeded into the left channel of the device to adhere to the fibrin in the center channel and the PDMS wafer as well as the glass (Figure 1b). HUVEC created an integrated endothelial monolayer extended in 3D 24 h postseeding (Figure 1c,d). Staining with PECAM‐1 (CD31) and VE‐Cadherin showed formation of strongly connected endothelial junctions (Figure 1c). The monolayer appeared to be stable for up to 5 days showing a high degree of barrier function.

To demonstrate the quality of the endothelial barrier, function of the left channel was perfused with fluorescently labeled 40 kDa dextran (Figure 1e). The dextran diffused slowly through the endothelial barrier, demonstrating a good degree of barrier function and ability to create chemical gradients within the device. While media, nutrients, and secreted factors were present throughout the device, any factor added into the right channel created concentration gradients over a span of a few hours allowing investigation into directed cell migration.

To assess extravasation rates, CD34^+^ HSC were seeded into the endothelial channel. After 24 h, some HSC were observed at the interface of the endothelial monolayer‐fibrin gel (Figure 1f, inset) and some had migrated away from monolayer fully embedded within the fibrin gel **(**Figure 1f, white arrow) indicating different stages of extravasation. An example of an HSC passing through endothelial cells while squeezing itself is shown in (Figure 1f, inset) suggesting the HSC apply forces to transmigrate through tight endothelial barrier.^44^, ^45^

To quantify extravasation rates, the images were segmented at the endothelial border, with a counting area extending 200 μm from either side of the endothelial interface (Figure S1a). The number of HSC, which had moved directly away from the endothelial interface into the gel channel, were counted and divided by the total number of HSC located within the counting area. Interestingly, assessment of the extravasation potential of CD34^+^ HSC of different origins (i.e., isolated from fetal liver, bone marrow, umbilical cord blood, and Lin^−^/CD3^−^/CD4^−^/CD8^−^ triple negative [TN] thymocytes) showed that these different HSC sources had similar intrinsic extravasation rates, with an average of 20% cells extravasated after 24 h (Figure 1g). Moreover, the intrinsic extravasation rate of CD34^+^ HSC from fetal liver sourced at different weeks postconception (wpc) stayed at the same levels, despite the concordant differences in developmental stage (Figure 1h).

### 2.2 Cytokines and TSC increase HSC extravasation rates

**FIGURE 2:**
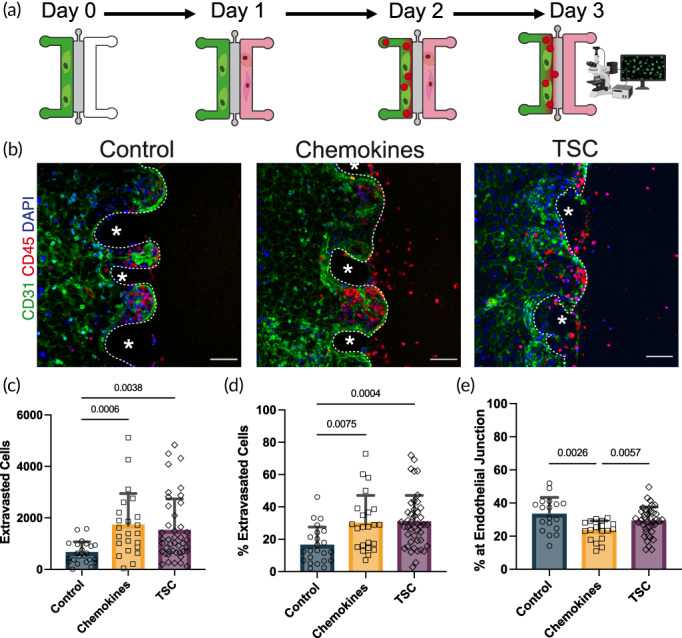
Experimental setup for investigating hematopoietic stem cell (HSC) extravasation under different conditions. (a) A schematic showing different stages of experiments: After establishment of integrated endothelial barrier either TEC and TIC were seeded into the right media channel or media added to the right channel; 24 h later, CD34+ HSC were added to the left channel and allowed to interact with endothelium for 24 h, before taking z‐stack confocal images for visualization and quantification of extravasation. (b) Examples of z‐projection images showing the spatial distribution of HSC (red) interacting with endothelium (green) under different conditions. Endothelial interface marked with dashed white line and polydimethylsiloxane (PDMS) pillars indicated with asterisks (*). Scale bars 100 μm. (c, d) The number of extravasated HSC in (c) and their extravasation rates in (d) for three conditions: control (human umbilical vein endothelial cells [HUVEC] and HSC alone, *n* = 23), chemokines (HUVEC, HSC, and exogenous CXCL12 and CCL25, *n* = 23), and TSC (HUVEC, HSC, and TEC and TIC, *n* = 44). Statistics represented are *p* values from Kruskal–Wallis tests. (e) There are significantly fewer HSC localized at the endothelial border in devices with exogenous chemokines than control devices. Statistics are *p* values from Kruskal–Wallis tests

Formation of a tight endothelial monolayer in microfluidic channels provides the opportunity to create cytokine gradients across endothelium. Furthermore, to understand the role that the thymus microenvironment plays in TSP recruitment, we took the advantage of in vitro expansion of human thymic epithelial (TEC) and interstitial (TIC) progenitors that demonstrated the capacity to attract HSC from bone marrow in vivo.^29^ Therefore, we set out to apply a cytokine gradient and incorporate up to four different cell types within our microdevice (Figure 2a). Twenty‐four hours after formation of the endothelium, we seeded 120 K TSC previously expanded in culture into the right channel or added exogenous chemokines (100 ng/ml of CXCL12 and CCL25) to the media in the right channel. Twenty‐four hours after seeding of TSC, ~30 K CD34^+^ fetal liver HSC were injected into the right channel and their extravasation rates were assessed after an additional 24 h (Figure 2b). An average of 5439 HSC localized within 200 μm on either side of the endothelial border (Figure S1c) and the experimental condition within the right channel did not affect HSC quantification (Figure S1d). We found that the presence of exogenous chemokines or TSC significantly increased (4‐ and 3‐fold, respectively) the number of extravasated HSC (Figure 2c). Consequently, the extravasation rate was also significantly increased when exogenous chemokines or TSC were present (Figure 2d).

Quantification of the number of nonextravasated HSC localized within 20 μm of endothelial border (Figure S1b) suggested that while control and TSC conditions has similar percentages of HSC accumulated close to the endothelial border (34% ± 8.6% and 29% ± 8.7%, respectively; Figure 2e), surprisingly, the chemokine condition had significantly less accumulation of HSC at the endothelial border (20% ± 6.7%; Figure 2e). These observations suggest that despite increased extravasation rates in both chemokine and TSC conditions, the chemokine condition induced different extravasation kinetics.

### 2.3 Impact of cellular origins

**FIGURE 3:**
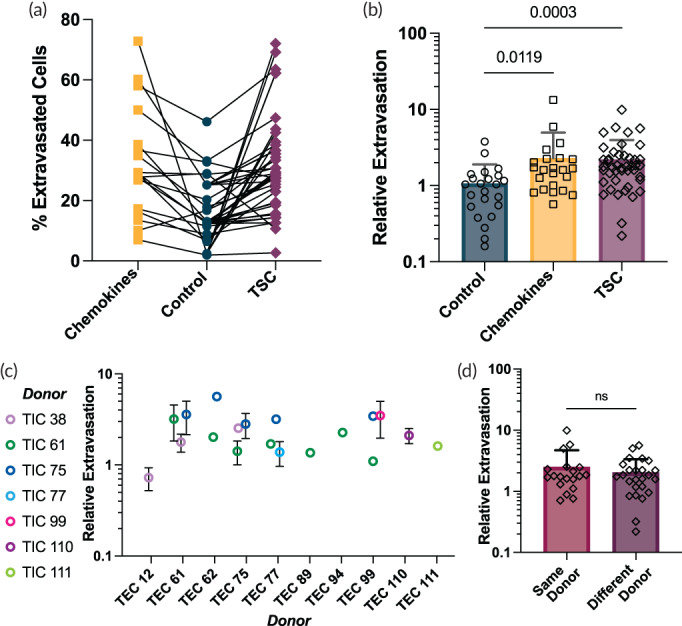
Effects of cell source, thymic epithelial cells (TEC), and thymic interstitial cells (TIC) on extravasation. (a) Extravasation rate matched by experimental replicates demonstrates variability derived from the hematopoietic stem cell (HSC). (b) To clarify biological variability, extravasation was plotted relative to extravasation rates in control devices by HSC source. Extravasation is significantly increased when exogenous chemokines or TSC are present. *p* values shown are the significant results of Kruskal–Wallis test. (c) The relative extravasation rate of HSC in TSC devices is sorted by TEC and colored by TIC. Experimental replicates are represented as the mean ± standard deviation. The heterogeneity of the recruitment capacity of the samples is reflected in the variance of the data, while demonstrating that TSC overall are exert a positive influence on extravasation rates. (d) Extravasation is not significantly different when TEC and TIC derived for the same donor are cultured together versus with stromal cells from different donors. No significant difference between groups (Mann–Whitney *p* = 0.6685)

Next, we investigated whether and how the observed enhanced HSC extravasation under chemokines or TSC co‐culture may depend on the different cell sources. Indeed, the source of primary stromal cells and their culture procedure may cause some variability in cellular behavior (Figure 3a). To dissect the role of HSC source on this variability, we normalized extravasation rates for each condition with respect to its own control extravasation rates (i.e., the same HSC source). Notably, we found that regardless of the HSC source extravasation rates were significantly increased under the influence of chemokines and TSC (3‐ and 5‐fold, respectively) (Figure 3b).

We also explored the influence of the source of thymic stroma by testing the extravasation of HSC co‐cultured with TSC that consists of mix culture of stromal TIC and TEC from different donors. While the cultivated TEC (Figure S2a–c,f,g) and TIC (Figure S2d–g) maintain their cellular identity in culture, the heterogenous nature of primary culture, and specifically the source of TSC (i.e., TIC and TEC from different donors) creates some degree of variability in the extravasation rates (Figure 3c). Nonetheless, the rates were consistently higher compared to controls (i.e., HSC extravasation with no stromal cells). Notably, incorporating TEC and TIC from different donors showed similar relative extravasation rates to those from the same donor (Figure 3d).

### 2.4 TEC and TIC act synergistically to recruit TSP

**FIGURE 4:**
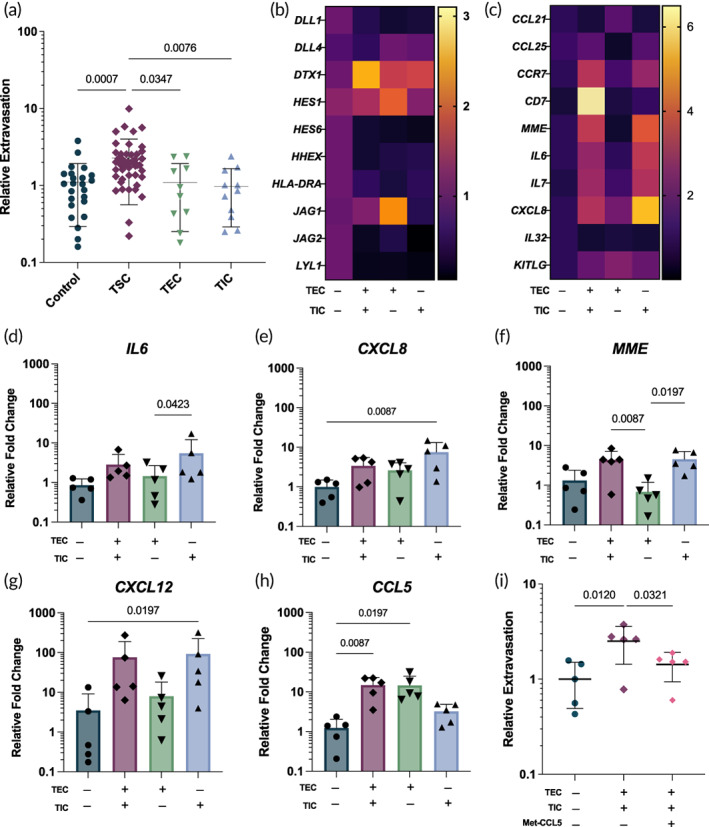
Gene expression of microdevices show cytokine expression and lineage commitment of hematopoietic stem cell (HSC). (a) There is significantly less extravasation of HSC when thymic epithelial cells (TEC) and thymic interstitial cells (TIC) are cultured in the devices alone rather than co‐cultured. *p* values are the significant results of Kruskal–Wallis test. (b) A heatmap of showing the relative fold change in gene expression for members of the Notch pathway, normalized to control (human umbilical vein endothelial cells [HUVEC] and HSC alone). (c) A heatmap of the relative fold change in gene expression of several cytokines and chemokines, normalized to control. (d) There is significantly different expression of IL‐6 (*IL6*) in devices with TIC rather than TEC, and devices with TIC alone tend to increase IL‐6 expression. (e) IL‐8 (*CXCL8*) expression is significantly increased by TIC. (f) TSC and TIC devices significantly increase CD10 (MME) expression compared with TEC alone (*n* = 5). (g) *CXCL12* is highly variable, but it tends to be driven by the TIC (e) *CCL5* expression is significantly increased in devices with TSC and TEC. (i) Pretreatment of TSC with a CCR5 antagonist (Met‐CCL5 100 ng/ml) led to significantly decreased extravasation compared to untreated devices (*n* = 5 per condition). (d–i) *p* values are significant results of Friedman test.

Motivated by the data suggesting that the presence of TSC increases HSC extravasation, we sought to expand the biological implications by assessing extravasation rates by seeding either TEC or TIC alone and found that the addition of TEC or TIC alone into the microdevices did not significantly change the extravasation rates compared to control. Surprisingly, combined culture of TEC and TIC showed significantly higher extravasation rates compared to individual TEC and TIC cultures suggesting that TEC and TIC act synergistically to recruit TSP (Figure 4a).

RNA from six independent experiments was collected from microdevices and analyzed by qPCR. Comparison of changes in gene expression to a number of chemokines, cytokines, as well as known indicators of lineage commitment, show that the cell types present in the device indeed influence the developing HSC. Investigation of known Notch ligands and downstream target genes showed that the Notch pathway was highly active. As the role of Notch has been known to play an important role in T‐cell commitment,^46^ we compared the gene expression of DLL1 and DLL4 in cultivated TSC and HUVEC (Figure S2h,i). This was likely driven by the presence of HUVEC, where tightly controlled Notch signaling is crucial for angiogenesis.^47^, ^48^ Likewise, expression of *DTX1*, a Notch positive regulator, increased when both TEC and TIC were introduced into the devices, thus suggesting that Notch activation was driven by the TSC as well (Figure 4b). Analysis of cytokine expression showed that several chemoattractant cytokines were expressed by combined TSC, such as *CXCL12*, *CCL5*, *IL7*, *CXCL8* (Figure 4c). IL‐7 (*IL7*), which is vital to thymocyte development and maturation, was upregulated in devices with TSC, though not significantly (Figure S3b and Figure 4c). Notably, SCF (*KITLG*), which is also crucial for HSC survival, was more expressed in devices repopulated by the thymic stroma than by the endothelial cells only (Figure S3a and Figure 4c). Pro‐inflammatory cytokines, such as IL‐6 (*IL6*), were significantly expressed when TSC or TIC alone were present (Figure 4d). IL‐8 (*CXCL8*) expression was high in all devices, but expression was significantly increased in devices seeded with TIC alone (Figure 4e). Crucially, the HSC tended to upregulate expression of ETP markers including *CD7* (Figure S3g), *CCR7* (Figure S3h), and CD10 (*MME*) (Figure 4h) when cultured in devices with thymic cells. *CXCL12*, used as one of the exogenous cytokines in the assay, was highly expressed in microdevices with TSC (Figure 4g). This expression was driven primarily by the TIC rather than the TEC (Figure S3e).

*CCL5* (RANTES) was also significantly expressed in devices containing TSC or TEC alone (Figure 4h and Figure S3f). A proinflammatory chemokine, CCL5 has been reported to regulate intrathymic migration in mice.^49^ To interrogate the role of CCL5 in HSC migration, TSC were pretreated with a CCR5 antagonist (Met‐CCL5, 100 ng/ml) before seeding and Met‐CCL5 was supplemented in the media of devices with TSC. Blocking CCR5/CCL5 signaling lead to decreased extravasation of HSC compared to the untreated condition (Figure 4i).

## 3 DISCUSSION

The properties of human thymus seeding progenitors, including the signals that induce their migration, as well as lineage commitment instructions have begun to be unraveled^50^ in recent years, particularly via transcriptomic methodologies.^14^, ^19^ However, the nature of these methods makes untangling the interactions across the multiple cell types within the thymus difficult. In addition, most in vitro assays relating to the thymus are focused on T‐cell development and rely on mouse cells, or mixed species cultures. We therefore sought to develop an assay to interrogate the interactions between human TSC, HSC, and endothelial cells at the first stages of thymus seeding.

Our microdevice‐based assay can assess the migration potential of HSC in response to thymus stroma. By separating the TSC or exogenous cytokines from the HSC and endothelial cells by a channel of fibrin, we were able to create an environment with a biochemical gradient, wherein more chemoattractant cytokines were on one side of the endothelium. The extravasation rate in the presence of TSC demonstrated that HSC migrate in a thymus specific manner recapitulating how HSC may migrate in vivo in response to signals from the thymus. This was further supported by the fact that different CD34^+^ HSC sources have similar extravasation rates.

The composite nature of this assay can be utilized to dissect the source of crucial signaling pathways. For instance, DLL1 signaling was highest in microdevices seeded with interstitial cells (TIC), but not when only epithelial cells (TEC) were present, suggesting that DLL1 signaling comes from the mesenchymal rather than the epithelial compartment. Likewise, DLL4 expression was high across all devices, suggesting that this expression was driven by HUVEC. As DLL1/DLL4/Notch1 expression and signaling are known to be important to T‐cell lineage commitment,^51^, ^52^ this suggests that the active process of thymic immigration may likewise be important for lineage commitment. Similarly, expression of CXCL12 appeared to be driven by the TIC in our system, whereas CCL25 expression has been shown to come from the TEC, this suggests that the compartments are likely to cooperate in vivo. Interestingly, CCL25 was not expressed by the thymic stromal cells in microdevices; however, the 48 h that the TSC were cultured in the devices may not have been sufficient time to induce its expression. Interleukin 7 (IL‐7) expression was high in the presence of TSC, and it is known that IL‐7 is necessary for thymocyte survival and has an important role in the differentiation of T cells; indeed, mutations in IL‐7 gene and its receptor have been implicated in severe combined immunodeficiency.^53^, ^54^ Interleukin 8, which is significantly expressed by TSC, is known to be expressed in humans by recent thymic emigrants,^55^ as well as microvascular endothelial cells^56^ but interestingly is not expressed in mouse thymus.^57^ As IL‐8 has also been implicated in T‐cell recruitment at inflammation sites,^58^ it may also play a parallel role in precursor homing within the human thymus.

CD10, a marker for the earliest thymic seeding progenitors,^19^ was significantly expressed in devices with TSC and TIC but not when only TEC were present, suggesting that TIC may play a crucial role in lineage commitment of thymocytes. As CD7 is a marker of lineage committed ETP, we were surprised to see expression in only 24 h, thus supporting that the thymic stroma may determine an inductive microenvironment for lymphoid lineages. Expression of these markers by HSC‐derived cells in microdevices with TSC suggests that T lineage commitment may occur via indirect interaction between thymic stroma and the HSC. The signals, which likely lead to this early commitment, may stem from the TIC rather than the TEC. While cross‐talk between TEC and thymocytes has been well described at least in mouse,^59^ the role of the mesenchymal cells of the thymus is emerging^60^ but has not been extensively investigated, highlighting the need to better understand this cellular compartment especially in human. In line with this, our data further support the important role of the nonepithelial stroma in thymus function.

While the intrathymic function of CCL5 (RANTES) is poorly understood, its expression has been implicated in myasthenia gravis, a thymoma‐related autoimmune disorder.^61^ On the contrary, the proinflammatory role of CCL5 (RANTES) has been well described in other contexts.^62^ Given the high expression of CCL5 by cells seeded within the microdevice, we used the assay to assess the role of CCL5 in HSC migration. Inhibition of CCL5/CCR5 signaling by a CCL5 antagonist within the microdevice decreased HSC migration suggesting a previously undescribed role of CCL5 in thymus seeding. This also demonstrated the usefulness of the assay for therapeutic screening.

In summary, our results show that cooperation among different stromal populations of the thymus, including endothelial, epithelial, and interstitial cells, is crucial for TSP seeding and lymphoid commitment not only in vivo but also in vitro. Deciphering the signals that recruit progenitors to the thymus is important for both furthering our understanding of human thymus development, but also has clinical implications, including for reconstitution interventions following HSCT gene therapies. By identifying the signals, which direct thymus seeding, we could develop novel approaches to improve T‐cell output and T‐cell repertoire longevity postintervention in the clinic.

## 4 MATERIALS AND METHODS

### 4.1 Human tissue

Postnatal thymi were donated by patients undergoing cardiothoracic surgery at the Great Ormond Street Hospital. A written informed consent was obtained from the patient parents or legally authorized representatives under ethical approval (REC No 15/YH/0334 and 07/Q0508/43‐06‐MI‐13 (B)). Human fetal livers were provided by the Joint MRC/Wellcome Trust Human Developmental Biology Resource (HDBR) under informed ethical consent with Research Tissue Bank ethical approval (REC No 08/H0712/34 + 5 and 08/H0906/21 + 5). Umbilical cord blood (CB) was obtained from normal full‐term deliveries after signed informed consent. CB sample collection was approved by the East London Ethical Committee (REC No 06/Q0604/110) and in accordance with the Declaration of Helsinki. Human bone marrow (BM) cells were commercially obtained (StemCell Technologies).

### 4.2 Cell culture

HUVEC were commercially obtained (Lonza) and cultured in microvascular endothelial media (EGM2‐MV, Lonza) in collagen‐treated cell culture flasks at 37°C, in 5% CO_2_ and 20% O_2_. Human thymus tissue was obtained from postnatal patients under written, informed consent. Thymic stromal cells were isolate as described by Campinoti et al., but briefly, TEC were isolated from tissue by mechanical and enzymatic dissociation to a single‐cell level and cultured on irradiated mouse fibroblasts (3T3) in culture media at 37°C, in 5% CO_2_ and 20% O_2_.^29^ TIC were isolated from thymus tissue via FACS or explant and expanded in culture media (Megacell, Sigma) at 37°C, in 5% CO_2_ and 5% O_2_.^29^

### 4.3 Microfluidic fabrication and assay

Molds for wafers were drafted in Autocad (CAD) and cut from 500 μm acrylic sheets with a laser cutter. The PDMS wafers were fabricated using the Sylgard 184 Silicone Elastomer Kit (Dow). The silicone elastomer base and curing agent were mixed at a ratio of 10:1, respectively. The PDMS was subsequently de‐gassed in a vacuum chamber for 30 min, gently poured into molds, and de‐gassed a second time for 30 min to allow air bubbles to rise out of the holes where the posts would be formed. The assembled molds were cured in the oven at 80°C for 2 h. After the PDMS polymerized, media ports were punched with a 2 mm biopsy tool and the hydrogel ports were punched with a 1 mm biopsy tool. PDMS wafers were sterilized at 120°C for 20 min, sonicated in 70% ethanol for 5 min, and bound to #1.5 glass coverslips in a plasma cleaner (HPT‐100, Henniker Plasma) at 50% power for 1 min. A final bake to ensure complete binding and hydrophobicity of the device was performed at 80°C overnight.

Fibrinogen (Sigma) was resuspended in Ca^−^/Mg^−^ PBS (GE) at 6 mg/ml. Fibrinogen was mixed with thrombin (Merck) in media in equal parts and added to central channel of device and allowed to polymerize at room temperature for 30 min in a humidity chamber. Meanwhile, HUVEC were detached from flasks using TrypLE (Gibco), washed, and counted. After fibrin polymerization, left media channel was coated with Matrigel (Corning) diluted 1:100 in media for 1 min. Channel was flushed with cold media and then 150,000 HUVEC in media were added. Normal media was added to right channel. Device was rested at a 90° angle for 30 min to allow HUVEC to sit down on fibrin and PDMS posts. After cells were given time to sit down, devices were placed in petri dishes and placed in a 37°C degree incubator at a 45° angle and incubated overnight in 5% CO_2_ and 20% O_2_. After 24 h, TEC and TIC were lifted using TrypLE, washed, and counted. Right media channels were coated with Matrigel as above. For devices with thymic stromal cells, 120,000 total cells were added to the right media channel in media. For chemokine devices, 100 ng/ml of huCXCL12 (Biolegend) and 100 ng/ml huCCL25 (Biolegend) were diluted in media and added to right media channel. Devices were returned to incubator with the petri dishes kept flat to allow the TSC to sit down overnight. HSC were thawed in 37°C water bath, washed, and resuspended in media for counting. Approximately 30,000 HSC in media were added to the left media channel. Right media channels were refreshed with media, supplemented as experimentally prescribed. Devices were returned to a 37°C degree incubator at a 45° angle and incubated for 24 h in 5% CO_2_ and 20% O_2_.

### 4.4 CCR5 antagonist

Recombinant human CCL5/Met‐RANTES (R&D Systems) was added to flasks with cultured TEC and TIC at 100 ng/ml 24 h before seeding microdevices. Sterile PBS was added to untreated flasks as a control. The 100 ng/ml of CCL5/Met‐RANTES was added to EGM2 media of experimental devices and assay continued as described above.

### 4.5 Immunofluorescence staining and image acquisition

Endothelial cells were stained with CD31 (Clone WM59, Biolegend) alone or co‐stained with VE‐Cadherin (Clone BV9, Biolegend). HSC were stained with CD45 (Clone HI30, Biolegend) alone or with CD34 (Clone 581, Biolegend). Hoechst 33342 (4082 S, NEB) was used to stain nuclei. Devices were imaged on an inverted Nikon confocal microscope with CSU‐W1 scanning unit after staining.

### 4.6 Assessment of barrier function

Twenty‐four hours or 72 h after seeding endothelial monolayer 40 kDa dextran labeled with TexasRed (Thermo Fisher) was resuspended in media and added to left channel of microfluidic device. Images were taken every 5 min for 2 h, or ever minute for 10 min. Dextran reaches right channel within 10 min and fully diffuses by 2 h.

### 4.7 Quantification of extravasation

Extravasation assay images were compiled in FIJI using a Max Projection of the 20 μm Z‐stacks.^63^ Images were then imported into QuPath‐0.2^64^ and the endothelial border was drawn based on CD31 expression using the magic wand tool. A counting area was created by merging 200 μm expansions from the endothelial border in either direction (Figure S1a). The endothelial junction area was calculated as 20 μm inwards from the endothelial border (Figure S1b). CD45^+^ cells were counted using the cell detection tool, based on CD45 expression thresholding. Cell counts in the counting area ranged from 73 to 16,219 cells (Figure S1c). Extravasated cells were considered those cells located within the gel channel, up to 200 μm from the endothelial border. To assess the extravasation rates, the number of extravasated cells was divided by the number of HSC located within the counting area. The culture condition in the right channel did not have a significant effect on the total number of cells counted (Figure S1d).

### 4.8 Gene expression by qPCR

RNA was extracted from microdevice via direct lysis. Subsequent RNA extraction was performed per manufacturer's instructions with the ReliaPrep RNA Cell Miniprep System (Promega Z6010) and eluted in nuclease‐free water and stored at −80°C. RNA concentration was quantified by Nanodrop and then reverse transcribed into cDNA using the GoScript Reverse Transcription Mix with Random Primers (Promega A2801) per the manufacturer's protocol. cDNA was diluted 1:5 in nuclease free water and stored at −20°C. qPCRs were performed using primers and FAM/TAMRA probes from IDT (Integrated DNA Technologies, USA) and PrecisionPLUS 2X qPCR MasterMix with Low ROX (PrimerDesign, UK) as per manufacturer's instructions. Relative gene expression was normalized to two housekeeping genes (β‐actin and TBP) and then benchmarked to control devices.

### 4.9 Statistics

The exact sample size (*n*) for each experiment is given as a discrete number and demonstrated graphically. Statistical analysis was performed using one‐way nonparametric ANOVA with post hoc analysis unless otherwise stated. Plots and graphs were generated with GraphPad Prism 9.

## AUTHOR CONTRIBUTIONS

**Sara Watson:** Conceptualization (equal); data curation (lead); formal analysis (lead); investigation (lead); methodology (lead); software (equal); validation (lead); visualization (lead); writing – original draft (lead); writing – review and editing (lead). **Yousef Javanmardi:** Formal analysis (supporting); investigation (supporting); methodology (supporting). **Luca Zanieri:** Investigation (supporting); methodology (supporting); resources (supporting). **Negar Shahreza:** Investigation (supporting); methodology (supporting). **Roberta Ragazzini:** Investigation (supporting); methodology (supporting); resources (supporting). **Paola Bonfanti:** Conceptualization (lead); data curation (supporting); formal analysis (equal); funding acquisition (lead); investigation (supporting); methodology (supporting); project administration (lead); resources (equal); software (supporting); supervision (lead); validation (supporting); visualization (supporting); writing – original draft (equal); writing – review and editing (equal). **Emad Moeendarbary:** Conceptualization (lead); data curation (supporting); formal analysis (supporting); funding acquisition (lead); investigation (supporting); methodology (lead); project administration (lead); resources (lead); software (supporting); supervision (lead); validation (supporting); visualization (supporting); writing – original draft (equal); writing – review and editing (equal).

## CONFLICT OF INTEREST

All authors declare that there are no conflicts of interest related to this work.

### PEER REVIEW

The peer review history for this article is available at https://publons.com/publon/10.1002/btm2.10454.

## Supporting information


**Supplemental Figure S1: Quantification of Extravasation. (a)** HSC were stained for CD45 expression and then counted via CD45 expression thresholding (red). The cells localized within 200 μm in either direction (left border yellow, right border cyan) of the endothelial border (right magenta) were counted. **(b)** The cells at the endothelial border are those located with 20 μm of the inside of the endothelial border, (shaded yellow). **(c)** An average of 5439 cells was ounted across 114 devices. **(d)** The number of HSC counted was not significantly different with regards to the type of media or cells plated in the right channel.
**Supplemental Figure 2: Characterization of thymic stromal cells during expansion. (a)‐(g)** Gene expression of cultivated TEC and TIC in culture, based on thymic stromal cell markers published by Campinoti et al.,^29^ which particularly characterizes a unique epithelial/mesenchymal population of TEC, which is capable of long‐term survival, expansion, and morphogenesis ex vivo. (c) *p = 0.0317, **p = 0.0092. (d) *p = 0.0309, **p = 0.0066. (f) *p = 0.0216 (g) *p 0.0425, **p = 0.0056, (h) **p = 0.0077. The gene expression of DLL1 **(h)** and DLL4 **(i)** in TEC, TIC and HUVEC is shown. *p = 0.0184.
Supplemental Figure 3: Characterization of TEC and TIC cultured in microdevices. (a)‐(f) Expression of cytokines and growth factors expressed by TEC and TIC cultured alone or together in microfluidic device for 48 h. *KITLG* (a) and *IL7* (b) were not significantly different but are crucial for HSC survival. *CXCL8* (c) and *IL6* (d) expression was not significantly different when cultured without the influence of the presence of HUVEC and CD34+ HSC. *CXCL12* (e) expression is significantly increased in TIC compared to TEC (*p = 0.0342). *CCL5* (f) expression is significantly increased in TEC compared to TIC (*p = 0.0342). (g) *CD7* was not expressed by HSC in all experiments (*n* = 3) but expression increased when thymic stroma was present within the devices (*p* = 0.0476, Student's T test). (h) The expression of CCR7, a receptor expressed on lymphocytes important for thymus homing, was increased in devices with TSC but not significantly (*p* = 0.0858). (e–f, h) *p* values are results of Friedman test.

## Data Availability

The data that support the findings of this study are available from the corresponding author upon reasonable request
