# Supplementary material for: Integrated role of human thymic stromal cells in hematopoietic stem cell extravasation
Source: Bioeng Transl Med. 2022 Nov 17;8(2):e10454. doi: 10.1002/btm2.10454 (PMC10013751; doi:10.1002/btm2.10454)
Supplement: Supplementary file 1 — Supplemental Figure S1: Quantification of Extravasation. (a) HSC were stained for CD45 expression and then counted via CD45 expression thresholding (red). The cells localized within 200 μm in either direction (left border yellow, right border cyan) of the endothelial border (right magenta) were counted. (b) The cells at the endothelial border are those located with 20 μm of the inside of the endothelial border, (shaded yellow). (c) An average of 5439 cells was ounted across 114 devices. (d) The number of HSC counted was not significantly different with regards to the type of media or cells plated in the right channel. Supplemental Figure 2: Characterization of thymic stromal cells during expansion. (a)‐(g) Gene expression of cultivated TEC and TIC in culture, based on thymic stromal cell markers published by Campinoti et al.,29 which particularly characterizes a unique epithelial/mesenchymal population of TEC, which is capable of long‐term survival, expansion, and morphogenesis ex vivo. (c) *p = 0.0317, **p = 0.0092. (d) *p = 0.0309, **p = 0.0066. (f) *p = 0.0216 (g) *p 0.0425, **p = 0.0056, (h) **p = 0.0077. The gene expression of DLL1 (h) and DLL4 (i) in TEC, TIC and HUVEC is shown. *p = 0.0184. Supplemental Figure 3: Characterization of TEC and TIC cultured in microdevices. (a)‐(f) Expression of cytokines and growth factors expressed by TEC and TIC cultured alone or together in microfluidic device for 48 h. KITLG (a) and IL7 (b) were not significantly different but are crucial for HSC survival. CXCL8 (c) and IL6 (d) expression was not significantly different when cultured without the influence of the presence of HUVEC and CD34+ HSC. CXCL12 (e) expression is significantly increased in TIC compared to TEC (*p = 0.0342). CCL5 (f) expression is significantly increased in TEC compared to TIC (*p = 0.0342). (g) CD7 was not expressed by HSC in all experiments (n = 3) but expression increased when thymic stroma was present within the devices (p = 0.0476, Student's T test [file BTM2-8-e10454-s001.docx]

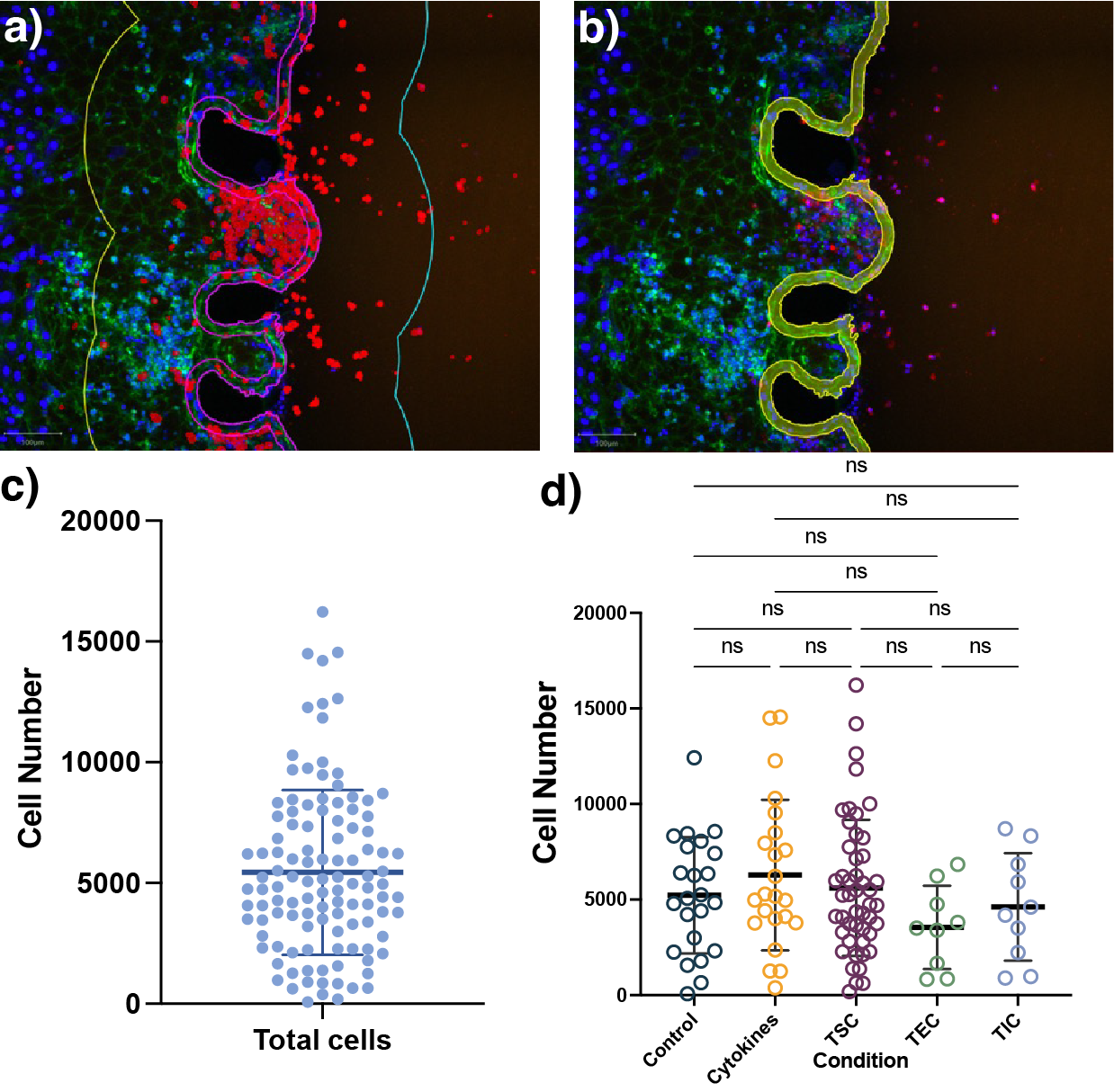


**Supplemental Figure 1:** **Quantification of Extravasation. (a)** HSC were stained for CD45 expression and then counted via CD45 expression thresholding (red). The cells localized within 200µm in either direction (left border yellow, right border cyan) of the endothelial border (right magenta) were counted. **(b)** The cells at the endothelial border are those located with 20µm of the inside of the endothelial border, (shaded yellow). **(c)** An average of 5439 cells were counted across 114 devices. **(d)** The number of HSC counted was not significantly different with regards to the type of media or cells plated in the right channel.

**
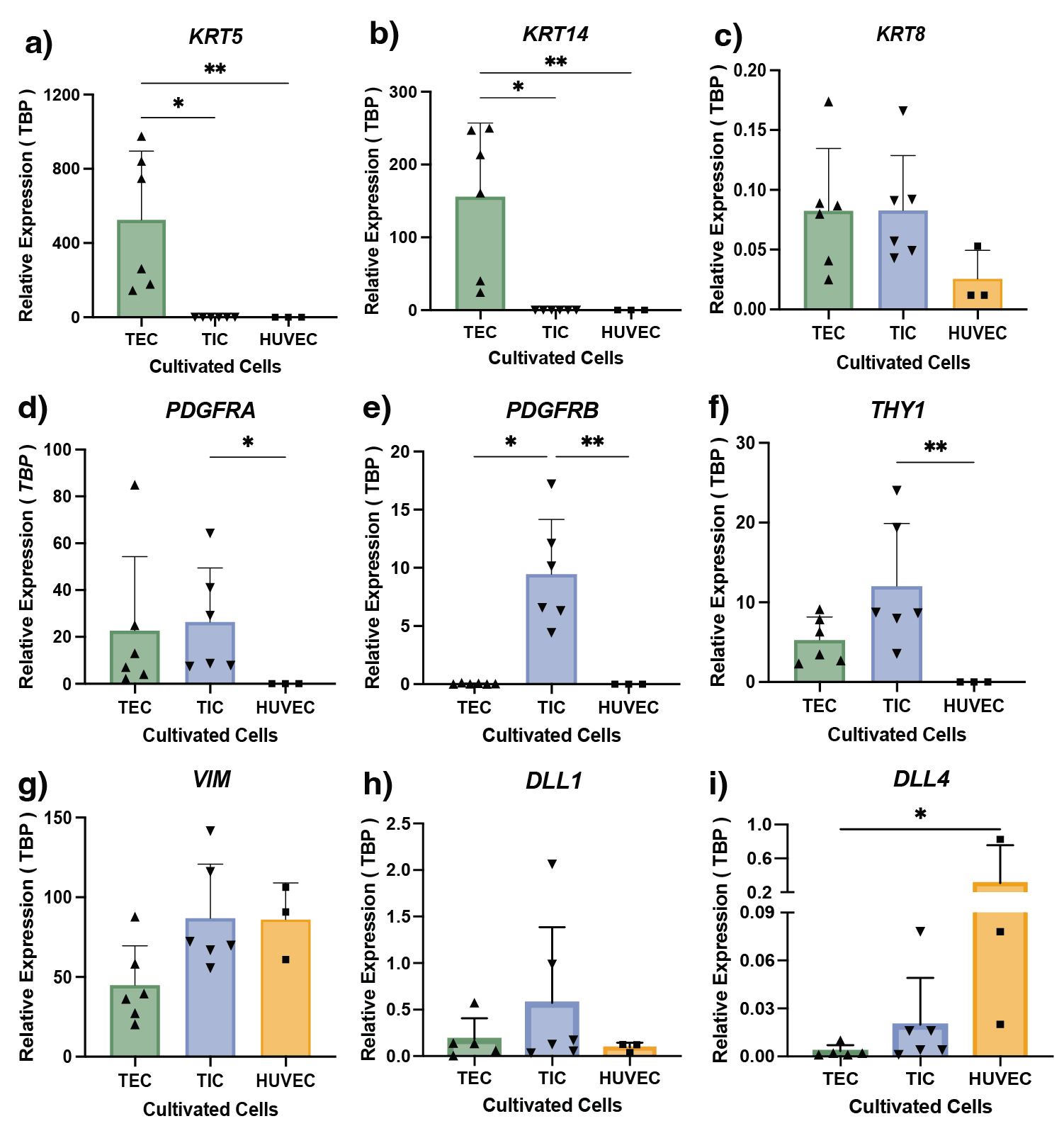
**

**Supplemental Figure 2:** **Characterization of thymic stromal cells during expansion. (a)-(g)** Gene expression of cultivated TEC and TIC in culture, based on thymic stromal cell markers published in Campinoti et al^29^, which particularly characterizes a unique epithelial/mesenchymal population of TEC which is capable of long term survival, expansion, and morphogenesis *ex vivo*. (c) *p = 0,0317, **p = 0,0092. (d) *p = 0.0309, **p = 0.0066. (f) *p = 0.0216 (g) *p 0.0425, **p = 0.0056, (h) ** p = 0.0077. The gene expression of DLL1 **(h)** and DLL4 **(i)** in TEC, TIC and HUVEC is shown. *p = 0.0184.


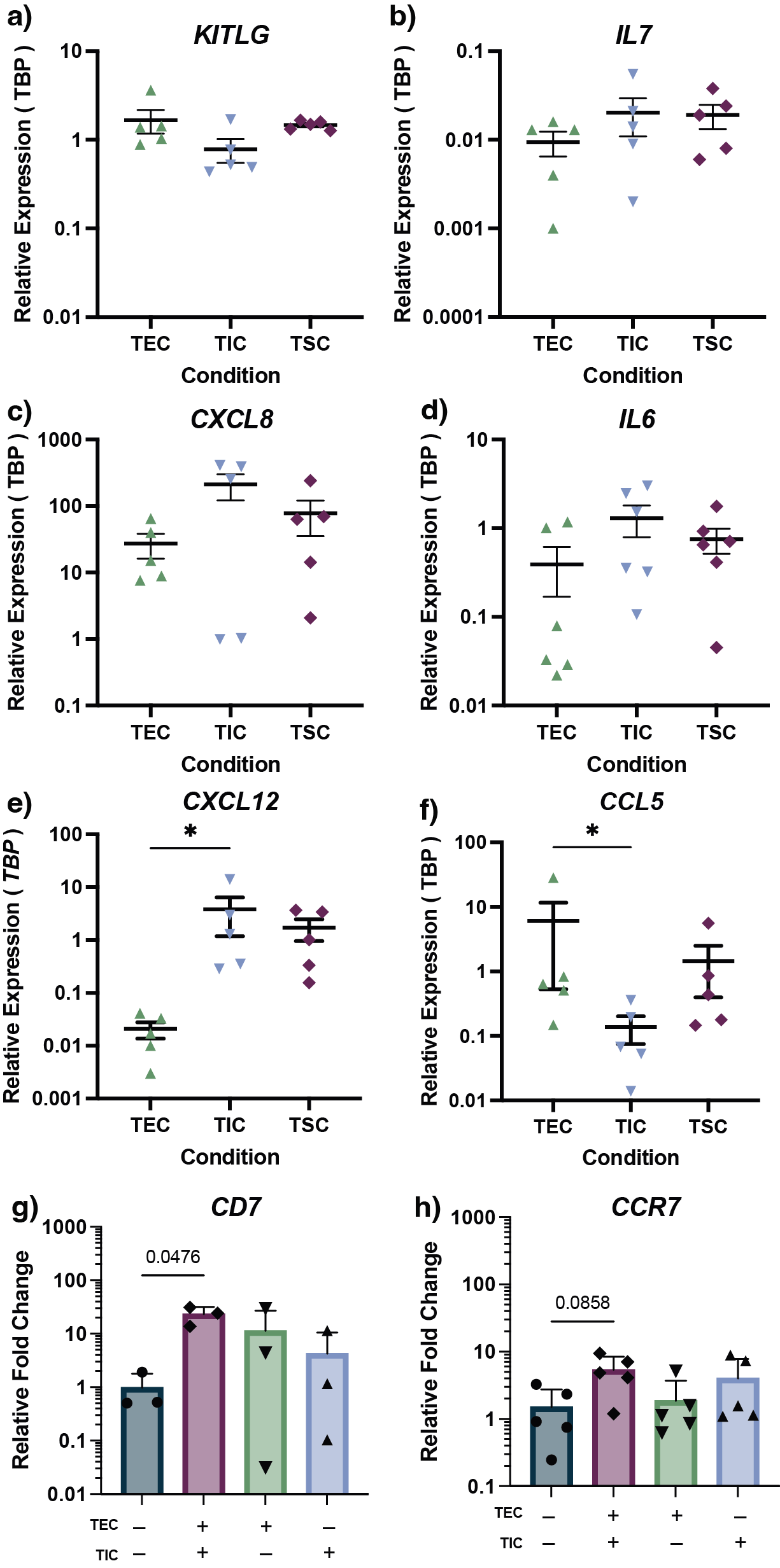


**Supplemental Figure 3:** **Characterization of TEC and TIC cultured in microdevices. (a)-(f)** Expression of cytokines and growth factors expressed by TEC and TIC cultured alone or together in microfluidic device for 48 hours. *KITLG* (a) and *IL7* (b) were not significantly different but are crucial for HSC survival. *CXCL8* (c) and *IL6* (d) expression was not significantly different when cultured without the influence of the presence of HUVEC and CD34+ HSC. *CXCL12* (e) expression is significantly increased in TIC compared to TEC (*p = 0.0342). *CCL5* (f) expression is significantly increased in TEC compared to TIC (*p = 0.0342). **(g)** *CD7* was not expressed by HSC in all experiments (n=3) but expression increased when thymic stroma was present within the devices (p=0.0476, Student’s T test). **(h)** the expression of CCR7, a receptor expressed on lymphocytes important for thymus homing, was increased in devices with TSC but not significantly (p = 0.0858). (e)-(f), (h) P values are results of Friedman test.
